# Myeloid Krüppel-Like Factor 2 Critically Regulates K/BxN Serum-Induced Arthritis

**DOI:** 10.3390/cells8080908

**Published:** 2019-08-16

**Authors:** Manjusri Das, Moonmoon Deb, Dipranjan Laha, Matthew Joseph, Suman Kanji, Reeva Aggarwal, O. Hans Iwenofu, Vincent J. Pompili, Wael Jarjour, Hiranmoy Das

**Affiliations:** 1 Department of Internal Medicine, Wexner Medical Center at The Ohio State University, Columbus, OH 43210, USA; 2 Department of Pharmaceutical Sciences, School of Pharmacy, Texas Tech University Health Sciences Center, Amarillo, TX 79106, USA; 3 Department of Pathology, College of Medicine, Wexner Medical Center at The Ohio State University, Columbus, OH 43210, USA

**Keywords:** rheumatoid arthritis, KLF2, inflammation, monocytes, osteoclasts, MMP9, conditional knockout mice

## Abstract

Rheumatoid arthritis (RA) is an immune-mediated inflammatory disease, and Krüppel-like factor 2 (KLF2) regulates immune cell activation and function. Herein, we show that in our experiments 50% global deficiency of KLF2 significantly elevated arthritic inflammation and pathogenesis, osteoclastic differentiation, matrix metalloproteinases (MMPs), and inflammatory cytokines in K/BxN serum-induced mice. The severities of RA pathogenesis, as well as the causative and resultant cellular and molecular factors, were further confirmed in monocyte-specific KLF2 deficient mice. In addition, induction of RA resulted in a decreased level of KLF2 in monocytes isolated from both mice and humans along with higher migration of activated monocytes to the RA sites in humans. Mechanistically, overexpression of KLF2 decreased the level of MMP9; conversely, knockdown of KLF2 increased MMP9 in monocytes along with enrichment of active histone marks and histone acetyltransferases on the MMP9 promoter region. These findings define the critical regulatory role of myeloid KLF2 in RA pathogenesis.

## 1. Introduction

Rheumatoid arthritis (RA) is the most common form of inflammatory arthritis affecting a significant portion of the population worldwide; in those affected it causes moderate to severe disability [1]. Immune dysregulation is critical in the pathophysiology of RA, in which progressive destruction of synovial joints, articular cartilage, and bones are observed [2,3]. Both innate and adaptive immune responses are critical in RA pathogenesis. The innate immune response includes immune complex-mediated complement activation; the adaptive immune responses are effected against auto-antigens and are comprised of post-translationally modified proteins, dysregulated cytokine networks, osteoclast and chondrocyte activation, and resident stromal cells that support RA disease progression [3,4,5]. Various metabolic and genetic factors also play important roles in RA pathogenesis [6,7]. In addition, genetic and epigenetic factors contribute to RA pathogenesis, making it a complex syndrome with a common clinical phenotype arising from diverse pathways and operating variably in individual patients [5]. 

Although various cell types contribute to RA pathogenesis, the important disease-initiating role of monocytes has been recognized [8]. Monocytes are augmented in number during active rheumatoid disease and their numbers are reduced after effective treatment; their plasticity, ability to respond rapidly to numerous stimuli, and secretion of proinflammatory effectors make them a very important cell type in RA pathogenesis [9,10,11]. Monocytes that migrate to the synovial joint tissues and differentiate into synoviocytes and macrophages produce numerous inflammatory mediators that interact with neighboring cells, immune cells, and extracellular matrix macromolecules, and directly influence their function [12,13]. Tissue macrophages also differentiate into osteoclasts, which are mainly responsible for subchondrial bone destruction in RA [14]. Osteoclasts are differentiated not only from the mature tissue macrophages but also from the immature cells of the monocyte-macrophage lineage, making monocytes central to the pathophysiology of RA [15]. RA pathogenesis and tissue destruction are induced by the higher level of matrix metalloproteinases (MMPs), such as MMP2, MMP3 and MMP9, which are abundant in RA tissues [16]. Several efforts have been made to find inhibitors for MMPs in order to more effectively treat RA [17]. Identifying a regulatory mechanism for monocyte activation and differentiation in RA and elucidating the way MMPs are regulated will lay the foundation for the development of future therapeutics that target specific molecules and thereby manage the disease.

The transcription factor Krüppel-like factor 2 (KLF2) plays an important role in the regulation of a variety of immune cells, including proinflammatory activation and function of monocytes [18,19,20,21], and myeloid cell polarization [22]. KLF2, also known as lung KLF, as it was first found in the adult mouse lung [23], plays a major regulatory role in hematopoietic cell biology, including cell quiescence, cell proliferation, differentiation, and survival [24]. It is evident that induction of KLF2 occurs during the maturation of single-positive T cells and it was found that it extinguishes after single positive T cell activation [24]. A quiescent T cell phenotype was noticed after forced overexpression of KLF2, whereas a spontaneously activated phenotype was visible in KLF2-null T cells [25]. Conversely, KLF2 deficiency in mice causes embryonic lethality due to leaky blood vessels and hemorrhaging [26].

Mechanistically, inflammatory gene activation requires interaction between co-activator molecules and regulatory transcription factors. The transcriptional co-activators P300 and P300/CBP-associated factor (PCAF) are histone acetyltransferases (HAT), that have been found to regulate numerous cell signaling pathways determining cell fate by acetylating both histone and non-histone proteins [27]. P300- and PCAF-mediated Histone 3 lysine 9 acetylation (H3K9Ac) and H4K5/8/12Ac enrichment promotes the binding of transcriptional initiation and elongation factors to the promoters of inflammatory genes [28,29]. Though the crucial role of epigenetic modulators in immune response has already been established, KLF2-associated regulation of epigenetic marks in RA is largely unknown. Moreover, it has yet to be established whether the regulatory role of KLF2 is critical in monocyte biology in the context of inflammatory RA.

The K/BxN serum-induced arthritis model is ideal for studying disease induction and pathogenesis for inflammatory arthritis. The K/BxN serum-induced arthritis model was developed by the Mathis/Benoist laboratory (Boston, Harvard Medical School) [30] by crossing T-cell receptor (TCR) transgenic KRN mice in C57BL/6 background with autoimmune-prone non-obese diabetic (NOD) mice that recognize a bovine ribonuclease peptide (RNase 43–56) presented by an I-Ak major histocompatibility complex (MHC) class II. The F1-generation, called K/BxN mice, developed severe arthritis within 4–5 weeks of age that rapidly progressed until mobility was significantly limited [30]. We received KRN mice from Dr. Mathis and followed their protocol to collect K/BxN serum from the F1 progeny; we then induced arthritis in KLF2 hemizygous mice as well as in monocyte-specific conditional KLF2 knockout mice, which were developed and characterized in the Jain laboratory (CWRU, Cleveland). In the current study, we used K/BxN serum-induced arthritis models to investigate the role of KLF2 in disease progression using various genetic models of KLF2 in mice.

## 2. Materials and Methods

### 2.1. Isolation and Culture of Monocytes from Murine Bone Marrow

Bone marrow (BM) cells were isolated from the femurs of 6–8 week-old KLF2^+/−^ (hemizygous), KLF2^−/−^ (monocyte-specific conditional knockout) and KLF2^+/+^ (wild type) mice of C57BL/6 background after flushing with phosphate-buffered saline (PBS). To make a single cell suspension and to remove red blood cells from BM, collected cells were filtered through a 70 μM nylon cell strainer (BD Labware, Franklin Lakes, NJ, USA) and treated with red blood cell (RBC) lysis buffer (Sigma Chemical Co., St Louis, MO, USA) to lyse red blood cells. Cells were then cultured in Dulbecco’s Modified Eagle’s Medium (DMEM) medium (Gibco, ThermoFisher, Grand Island, NY, USA), supplemented with 10% fetal bovine serum, 2 mM l-glutamine, 100 U/mL penicillin, and 100 μg/mL streptomycin (all from Gibco), at 37 °C in a 5% CO_2_ atmosphere for further stimulation or subjected to osteoclastic differentiation.

### 2.2. Isolation of Human Monocytes

Fresh human peripheral blood (*n* = 3) from healthy donors and from active RA patients (deidentified) was collected after institutional review board (IRB) approval and written consent from donors from The Ohio State University Medical Center, and processed following an earlier-described protocol [18,31]. In, brief, peripheral blood mononuclear cells were isolated from 40 mL of freshly collected blood using Ficoll-Paque density centrifugation. After removing platelets from the peripheral blood mononuclear cell (PBMC) by washing with PBS, CD14^+^ cells were isolated using an AutoMACS device, and magnetic bead conjugated CD14^+^ antibody and reagents (all from Miltenyi Biotec, San Diego, CA, USA) following an earlier-established protocol [18,19]. Isolated CD14^+^ cells were used for total RNA was extraction and quantitative reverse transcriptase polymerase chain reaction (RT-PCR) analysis.

### 2.3. Real Time RT-PCR Analysis

Total RNA was isolated from BM-derived monocytes from KLF2^+/−^, KLF2^−/−^ and KLF2^+/+^ mice, as well as from human peripheral blood-derived monocytes both from healthy donors and active RA patients using a RNeasy Kit (Qiagen, Thermo-Fisher). One microgram of RNA was used for synthesis of cDNA using an oligo dT (Invitrogen, Thermo-Fisher) primer. Real-time RT-PCR was performed using one micro liter of cDNA for the gene specific primers such as KLF2, IL-1β, IL-6, TNFα, CCL3, MCP-1, IL-10, MMP9, MMP13 and GAPDH (murine); KLF2, TNFα, MCP-1, MMP1, MMP9, MMP13 and GAPDH (human) using a standard SYBR green Taqman protocol and real-time PCR machine (Stratagene, MX3000P, Santa Clara, CA, USA). Relative fold-expression levels of the stated genes were measured considering respective unstimulated cells, wild type (WT) cells, or healthy controls as basal levels. Experiments were performed in triplicate and were repeated at least three times.

### 2.4. Osteoclast Differentiation and TRAP Staining

To determine the role of KLF2 in osteoclastic differentiation, bone marrow cells collected from various murine genotypes were subjected to induced osteoclastic differentiation following an established protocol [19]. In, brief, bone marrow cells were cultured overnight at 37 °C incubator with 5% CO_2_ in minimum essential medium (αMEM) containing 10% heat inactivated fetal bovine serum in the presence of 20 ng/mL macrophage colony stimulating factor (M-CSF) (R & D Systems, Minneapolis, MN, USA). The next day, non-adherent cells were collected and incubated for an additional 6 days in αMEM medium with 20 ng/mL M-CSF, and 50 ng/mL glutathione-S transferase conjugated receptor activator of nuclear factor-κB ligand (GST-RANKL) [32]. Fresh medium was replaced in every alternate day. At day 3 and 6 of differentiation, the cells were stained for tartrate-resistant acid phosphatase (TRAP) staining using an acid phosphatase, leukocyte TRAP staining kit (Sigma Aldrich) and they were viewed and imaged with a fluorescence microscope (Nikon, Axioplan2, Carl Zeiss). After TRAP staining, TRAP-positive multinucleated cells (3 nuclei, 4′,6-Diamidino-2-Phenylindole, DAPI was used for nuclear staining) were counted as osteoclast-like cells.

### 2.5. Osteoclast Immunostaining

Osteoclasts were cultured on either glass coverslips or on thin ivory slices and were fixed at various time points of culture with 1% formaldehyde in pH 6.5 (30 min at room temp) in stabilization buffer, and subsequently fixed and permeabilized with 2% formaldehyde, 0.2% Triton X-100, and 0.5% deoxycholate in the same stabilization buffer. Cells were stained with nuclear factor of activated T cells 1 (NFATc1), the vacuolar adenosine triphosphate synthase (VATPase), matrix metalloproteinase (MMP)13 and MMP9 specific antibodies as well as co-stained with Actin for cytoskeleton structure and visualized using a Zeiss confocal microscope (Campus Microscopy and Imaging Facility, The Ohio State University) following earlier-established protocol and antibodies [32]. Images were captured digitally using Axio vision software (Nikon, Axioplan2, Carl Zeiss). Bone resorptions by cultured osteoclasts were assessed on ivory slices by gently removing osteoclasts with cotton swabs followed by washing with water. The ivory slices were then stained with hematoxylin stain for 5 min at room temperature and excess stain was removed by washing with water; pits were then imaged with a confocal microscope as mentioned above.

### 2.6. Induction of Arthritis in Mice

All animal experiments were performed after institutional animal care and use committee (IACUC) approvals from the Ohio State University (IACUC # 2008A0191) and Texas Tech University Health Sciences Center (IACUC # 16038). To collect the arthritic serum, K/BxN mice were generated by crossing KRN, TCR-transgenic B6 mice (kind gift from Dr. Diane Mathis, Harvard Medical School, Boston, MA) with NOD mice (Jackson Laboratory, Bar Harbor, ME) following established protocol [30]. K/BxN serum was collected from 8-week-old arthritic K/BxN mice and pooled for each experiment. Each group of mice (C57BL/6 background 6–8 weeks old, female) for KLF2^+/−^ (*n* = 10), KLF2^−/−^ (*n* = 10) and KLF2^+/+^ (*n* = 20) was induced by intraperitoneal injection of 150 μL of K/BxN serum on days 0 and 2 following earlier-established protocol [33]. Mice were observed and ankle thickness was measured every day, and finally mice were sacrificed on day 8 following first injection of K/BxN serum. Hind limbs were harvested and part was subjected to histopahological analysis; another part was used for BM isolation for osteoclastic differentiation, RNA isolation, and protein isolation.

### 2.7. Histological Assessment of Arthritis

Arthritis was assessed by histological examination, as described in an earlier paper [19], with some modifications. Knee joints were exposed by removal of the overlying skin and then excised. Limbs were fixed in periodate-lysine-paraformaldehyde for overnight and decalcified in 10% ethylene diamine tetra acetic acid (EDTA), (BDH Chemicals, Victoria, Australia) and 7.5% polyvinylpyrolidone (Sigma) in Tris buffer (pH 6.95) for 7–10 days. After decalcification bones were processed for paraffin embedding. Tissues were sectioned with 5 μm thickness, placed on aminoalkylsilane-coated slides, and stained for routine histology with hematoxylin and eosin. Five defined pathologic features were graded for severity from 0 (normal) to 5 (severe), according to established protocol [34], and in a blinded manner. Soft tissue inflammation, assessed in the infrapatellar fat pads, joint capsule, and the area adjacent to the periosteal sheath, was graded according to the extent of cellular infiltration and angiogenesis. Joint space exudate was identified as leukocytes scattered discretely or in aggregates in the joint space. Synovitis was defined as hyperplasia of the synovium, but did not include pannus formation. Pannus was defined as hypertrophic synovial tissue forming a tight junction with the articular surface. Evaluation of cartilage and bone damage was based on loss of cartilage matrix, disruption and loss of cartilage surface, and the extent and depth of subchondral bone erosion [35]. A trained pathologist at the Department of Pathology, The Ohio State University Medical Center performed the histomorphometric analyses from all hematoxylin and eosin (H&E) sections.

### 2.8. Western Blotting

Total protein analysis was performed using the standard western blot method with equal amounts of proteins isolated from BM cells from KLF2^−/−^ and KLF2^+/+^ mice in the presence or absence of induced arthritis through fractionated sodium dodecyl sulfate (SDS)-polyacrylamide gel electrophoresis. Western blot analysis was performed using MMP9, MMP13, and GAPDH antibodies (all from Cell Signaling Technology, Danvers, MA, USA).

### 2.9. Immunohistochemistry for Human Samples

For identification of synovial macrophages both pathological and non-pathological control human tissues (deidentified) were obtained from the The Ohio State University Medical Center (OSUMC) clinic and from pathology department after the institutional review board (IRB) approval from The Ohio State University Wexner Medical Center (IRB # 201280195). Deparaffinized sections were incubated with specific mAb against CD68, and subsequently stained for 1 h using a peroxidase-conjugated rabbit anti-rat immunoglobin G (IgG) (Dako, Carpinteria, CA, USA). Endogenous peroxidase was blocked with 0.3% H_2_O_2_ (30% weight/volume; Sigma) in methanol. Peroxidase activity was demonstrated by incubation with in 3, 39-diaminobenzidine tetrahydrochloride (DAB; Sigma)-H_2_O_2_ solution. A peroxidase-conjugated rabbit anti-mouse IgG was incubated for 1 h, and color was developed with DAB-H_2_O_2_ solution, as described above, as well as by using a 5-bromo-4-chloro-3-indolyl-phosphate (BCIP)/(NBT) nitroblue tetrazolium substrate detection kit (Zymed Laboratories, South San Francisco, CA, USA). Sections were viewed and imaged with a microscope with a digital camera attachment (Nikon, Axioplan2, Carl Zeiss).

### 2.10. Overexpression and Knockdown of KLF2

KLF2 overexpression was induced in RAW264.7 cells, using control (Ad-GFP) or KLF2 (Ad-KLF2) virus in the presence of polybrene (final concentration, 8 ng/mL), as described in our earlier study [18]. Conversely, KLF2 knockdown was performed using mouse KLF2 sequence-specific siRNA and non-specific control using 60 nmol/L concentrations as described earlier [18].

### 2.11. Chromatin Immunoprecipitation (ChIP) and Quantitative RT-PCR

ChIP analysis was performed using Imprint^®^ Chromatin Immunoprecipitation Kit (Sigma). Briefly, after chromatin cross-linking with 1% formaldehyde and DNA shearing, chromatin-protein complexes were immunoprecipitated from siRNA nonspecific (siNS), siKLF2 and untreated RAW264.7 cells with antibodies against H3K9Ac (Millipore Sigma, 07-352), H4K8Ac (Millipore Sigma, 07-328), P300 (Millipore Sigma, 05-257) and PCAF (Cell Signaling, 3378S). Antibody against goat IgG (Abcam, ab1791) was used as a negative control. Quantitative RT-PCR analysis was performed with the primers described (in Supplementary Table S1 and Figure S1). Values obtained from the ChIP assay were normalized to the background obtained from the precipitation with a non-specific antibody. Percentage (%) of input was analyzed by following standard formula. Each experiment was performed in triplicate at least three times.

### 2.12. Statistical Analysis

Values were expressed as mean ± standard error of mean (SEM) and statistical analysis was performed by analysis of variance (ANOVA). Student’s *t*-test was performed and the results were considered significant when *p* values were <0.05.

## 3. Results

### 3.1. Effect of K/BxN Serum-Induced Arthritis in KLF2 Hemizygous Mice

**Figure 1:**
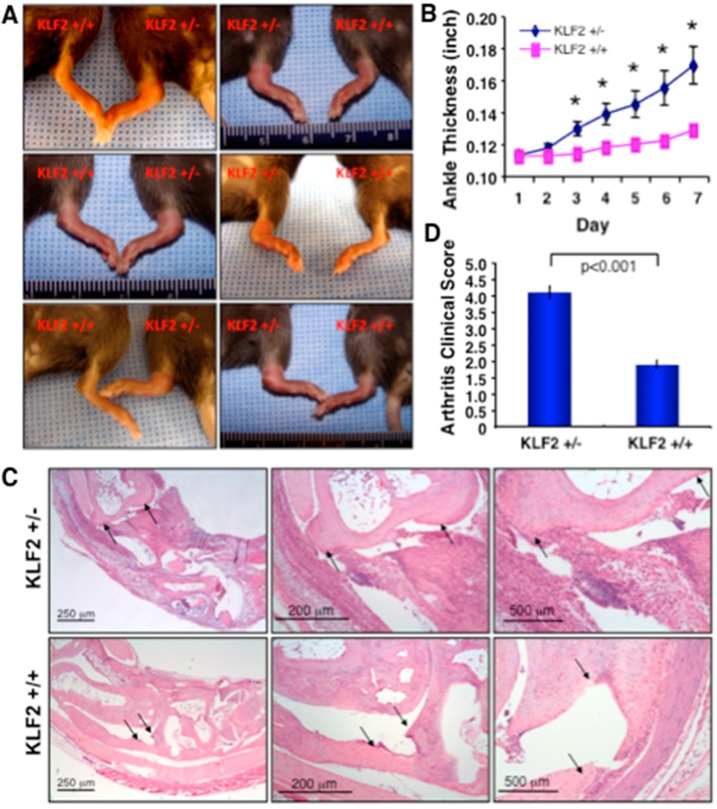
K/BxN serum-induced arthritic pathogenesis was elevated in Krüppel-like factor 2 (KLF2) hemizygous mice. (**A**) Acute arthritis was induced in KLF2^+/−^ and KLF2^+/+^ mice (C57BL/6 background) using K/BxN serum and inflammation and severity of pathogenesis in morphology were assessed. (**B**) Ankle thickness was measured during rheumatoid arthritis (RA) pathogenesis and graphically presented. (**C**) Hematoxylin and eosin (H&E) staining was performed to the joint tissue sections, which were evaluated for inflammation and bone and cartilage damage (arrow heads, upper panels). (**D**) Four mice in each group (KLF2^+/−^ and KLF2^+/+^), and three sections from each mouse were evaluated for assessment of clinical scores (five points were considered for scoring) and presented graphically.

The study of arthritic pathogenesis and the associated mechanisms governing inflammation was performed using a K/BxN serum-induced arthritis model, as inflammation and cellular mechanisms could be studied without interfering with joint tissues during induction of disease and pathogenesis. To evaluate the role of KLF2 in the regulation of arthritic inflammation and osteoclastic differentiation, we first assessed inflammatory response in KLF2^+/−^ mice (as KLF2 homozygous deficiency is embryonic lethal) after arthritic induction. KLF2^+/−^ mice developed severe paw swelling and joint inflammation after arthritis induction, whereas KLF2^+/+^ (WT) mice showed very mild paw and joint inflammation (Figure 1A). Ankle swelling was measured every day for 7 days and a statistically significant increase (*p* < 0.05) in ankle thickness in KLF2^+/−^ mice compared to KLF2^+/+^ mice was observed (Figure 1B). Mice were then sacrificed and the limbs were either formalin fixed, decalcified, paraffin blocked and sectioned, or used for bone marrow harvest. Hematoxylin and eosin (H&E) staining was performed on the sections. Joints (ankle to toe) were evaluated for damage and inflammation. When evaluated with histopathological parameters, KLF2^+/−^ mice showed significantly (*p* < 0.001) advanced pathogenesis (clinical scores) in terms of synovial inflammation and cartilage and bone erosion, compared to KLF2^+/+^ mice (Figure 1C,D). Therefore, morphological and histopathological analyses provide evidence of heightened arthritic pathogenesis in KLF2^+/−^ mice compared to KLF2^+/+^ mice.

### 3.2. Effect of KLF2 Hemizygosity on Osteoclast Differentiation and Maturation

**Figure 2:**
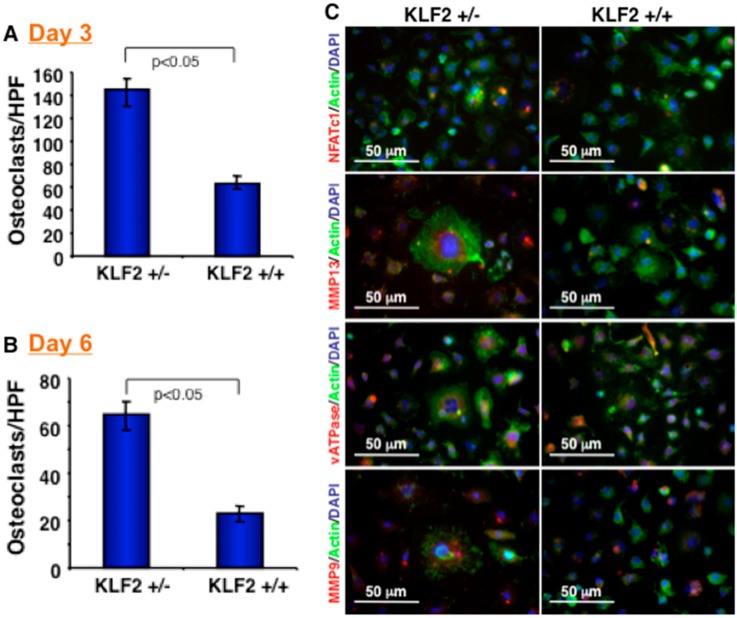
KLF2 hemizygocity enhanced osteoclast differentiation and maturation. (**A**) Osteoclast precursor cells were harvested from femurs of KLF2^+/−^ and KLF2^+/+^ mice after termination of K/BxN serum-induced arthritis, and osteoclasts were induced to differentiate. After tartrate-resistant acid phosphatase (TRAP) staining, the number of differentiated osteoclasts were measured and graphically presented at day 3. (**B**) Number of differentiated osteoclasts were measured and graphically presented at day 6. (**C**) Differentiated osteoclasts were stained for various osteoclast-related markers.

We next assessed the effect of KLF2 hemizygosity on osteoclast precursor cells after K/BxN serum-induced arthritis development. After 7 days of arthritis induction, osteoclast precursor cells were harvested from the bone marrow of the KLF2^+/−^ and KLF2^+/+^ mice. Osteoclastic differentiation was assessed on days 3 and 6 after induction of bone marrow precursor cells using TRAP staining. We observed a significantly increased number of osteoclasts differentiated from precursor cells isolated from the bone marrow of KLF2^+/−^ mice compared to KLF2^+/+^ mice. Notably, a higher number of nuclei were present in differentiated osteoclasts derived from KLF2^+/−^ mice compared to KLF2^+/+^ mice on day 3 (Figure 2A) and day 6 (Figure 2B) of differentiation. In addition, we evaluated the expression of relevant molecules in differentiated cells, such as NFATc1, MMP13, vATPase, and MMP9, which are important in the process of osteoclastogenesis. It is known that NFATc1 is the master transcription factor for osteoclast differentiation [36]. MMPs, on the other hand, are a family of proteolytic enzymes involved in the degradation of the extracellular matrix of various tissues, including bone; MMP13 is reported to be upregulated during osteoclast differentiation [37]. Reports also confirmed the role of MMP13 by knocking down this molecule, which drastically reduced bone destruction [38]. Because of its vital role in providing cellular machinery to osteoclasts during bone resorption, the vATPase complex is important in pathological bone diseases, including RA [39,40]. These molecules, therefore, are considered highly relevant to the process of osteoclastic differentiation. We found that expression of NFATc1, MMP13 and vATPase molecules in differentiated osteoclasts isolated from the bone marrow cells of arthritic mice was enhanced in KLF2^+/−^ mice compared to KLF2^+/+^ mice (Figure 2C).

### 3.3. Expression of Molecules in Bone Marrow after Induced Arthritis

**Figure 3:**
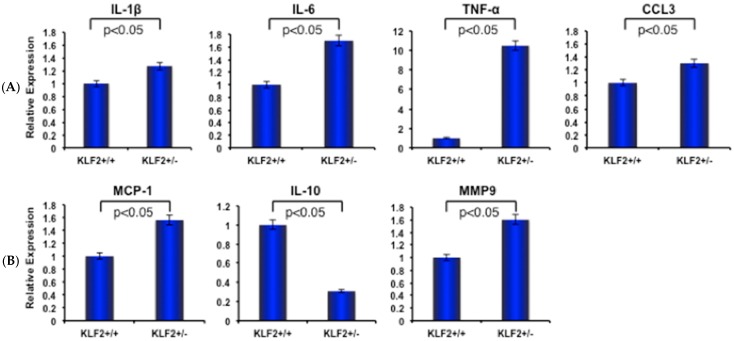
Inflammatory molecules are elevated in bone marrow cells of KLF2^+/−^ mice after induced arthritis. (**A**) Quantitative real-time PCR was performed to determine relative expression of various arthritis-related factors in bone marrow from KLF2^+/−^ and KLF2^+/+^ mice after development of K/BxN serum-induced arthritis. (**B**) The levels of matrix metalloproteinases (MMPs) were assessed in bone marrow from KLF2^+/−^ and KLF2^+/+^ mice after development of K/BxN serum-induced arthritis; mice without arthritis were used as controls.

Elevated expression of pro-inflammatory molecules correlates with increased severity of RA. IL-1β, one of the prototypic pro-inflammatory cytokines, plays key roles in both acute and chronic inflammatory, autoimmune disorders, and causes pathophysiological changes in RA [41]. IL-6 also has prominent effects in the development of chronic inflammation by mediating the regulation of trans-signaling intermediary factors that are involved in inflammatory mechanisms [42]. In addition, tumor necrosis factor-α (TNF-α) plays a pivotal role in regulating the inflammatory response in rheumatoid synovitis by controlling the production of other pro-inflammatory cytokines, including IL-6 [43]. On the other hand, IL-10 is an anti-inflammatory cytokine; in its absence, the pro-inflammatory response is augmented [44]. Several chemokines, such as chemokine ligand 3 (CCL3) and monocyte chemoattractant protein-1 (MCP1), also have a prominent role in RA progression and are present at high levels in the synovial tissue and fluids of RA patients. An increased level of MCP-1 was significantly correlated with levels of IL-6 in the culture supernatants of RA synovia, whereas CCL3 was also reported to be higher in the synovial fluid of RA patients [45,46]. As these molecules are all demonstrably important in the process of RA progression, we sought to determine how the expression levels of these molecules are changed upon induction of arthritis in KLF2^+/−^ mice. We found that expression levels of IL-1β, IL-6, TNFα, CCL3 and MCP-1 molecules were significantly (*p* < 0.05) increased after induction of arthritis in the bone marrow of KLF2^+/−^ mice compared to KLF2^+/+^ mice. Concomitantly, we saw a decreased level of IL-10 in KLF2^+/−^ mice compared to KLF2^+/+^ mice. In addition, the expression level of MMP9 was much higher in induced arthritic KLF2^+/−^ mice compared to KLF2^+/+^ mice (Figure 3).

### 3.4. Effect of Monocyte-Specific Conditional KLF2 Knock Out on K/BxN Serum-Induced Arthritic Pathogenesis

**Figure 4:**
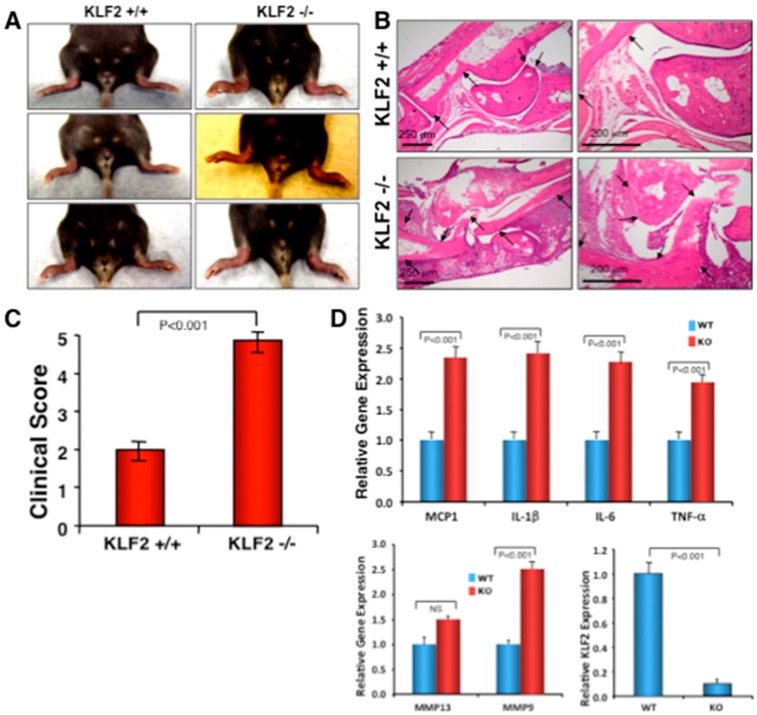
Monocyte-specific conditional KLF2 knock out enhances K/BxN serum-induced arthritic pathogenesis. The effect of K/BxN serum-induced arthritis was assessed in myeloid specific conditional KLF2 knockout mice. (**A**) Representative morphologies of arthritic inflammation in hind limbs. (**B**) Histopathological images of bone and joints after H&E staining. (**C**) Measured clinical scores presented graphically. (**D**) The relative expression of various arthritis-related factors in bone marrow of KLF2^−/−^ and KLF2^+/+^ mice after development of K/BxN serum-induced arthritis.

To determine if monocyte-specific KLF2 is indeed of critical importance for RA pathogenesis, K/BxN serum-induced arthritic induction was performed in monocyte-specific conditional KLF2 knockout mice. As shown in Figure 4A, visibly noticeable arthritic inflammation of limbs, severe joint stiffness, and impairment of movement were observed in KLF2^−/−^ mice compared to the KLF2^+/+^ mice, in which minimal swelling and distortion of ankle joints were observed and there was no impairment of movement. However, there were no significant changes in body weight between the KLF2^−/−^ and KLF2^+/+^ groups. Next, we verified the changes at the tissue level of ankle joints and toes in both KLF2^−/−^ and KLF2^+/+^ mice using histopathological microscopic studies. Characteristic severe hyperplasia of the synovial membrane, synovial thickening, pronounced progression of the disease, formation of a pannus, and massive production of fibrous tissue were observed in the ankle joints of the KLF2^−/−^ mice compared to KLF2^+/+^ mice (Figure 4B). These cases of severe cartilage and subchondral bone erosion observed in the joints of KLF2^−/−^ mice were associated with impaired movement. Clinical scores (five point) for the arthritic damage in KLF2 knockout mice were significantly higher (*p* < 0.001) than those of the KLF2^+/+^ mice (Figure 4C). Hence, these data support the assertion that monocyte-specific KLF2 is important in the regulation of K/BxN serum-induced arthritis.

To determine the role of monocytic cells in severe ankle inflammation and in the arthritic pathogenesis of KLF2^−/−^ mice, we isolated bone marrow cells and analyzed inflammation-related molecular expression along with osteoclastogenesis. Analysis using real-time PCR revealed that significantly increased (*p* < 0.001) levels of inflammatory molecules such as MCP-1, IL-1β, IL-6, TNF-α, and MMP9 were observed in KLF2^−/−^ mice compared to KLF2^+/+^ mice (Figure 4D). We did not see any significant difference in the level of MMP13 expression, although the level was slightly elevated in KLF2^−/−^ mice compared to KLF2^+/+^ mice. The expression level of KLF2 was reduced to almost 10% in bone marrow monocytic cells after conditional knockdown of the KLF2 gene (Figure 4D).

### 3.5. Effect of Monocyte-Specific KLF2 Deficiency on Osteoclast Differentiation and Function

**Figure 5:**
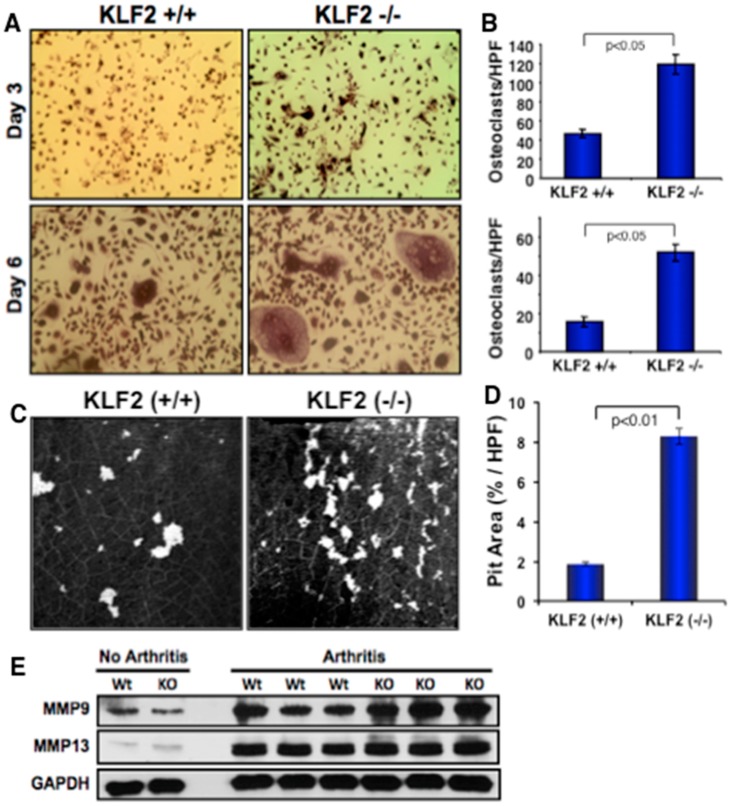
Monocyte-specific KLF2 deficiency enhanced osteoclast differentiation and MMP expression. (**A**) Differentiation of osteoclasts was evaluated on days 3 and 6 in bone marrow cells harvested from femurs of KLF2^−/−^ and KLF2^+/+^ mice upon K/BxN serum-induced arthritis development using TRAP staining. (**B**) The number of differentiated osteoclasts were measured and graphically presented at days 3 and 6. (**C**) Osteoclast functionality was evaluated using a pit-forming assay on ivory slices and using bone marrow cells harvested from femurs of KLF2^−/−^ and KLF2^+/+^ mice after K/BxN serum-induced arthritis development. (**D**) Evaluated values of pit area/high power field (HPF) are shown graphically. (**E**) Western blot analysis revealed that higher levels of MMP9 and MMP13 were present in bone marrow cells isolated from KLF2^−/−^ mice compared to the KLF2^+/+^ mice upon development of K/BxN serum-induced arthritis.

To determine the additional cellular and molecular mechanisms associated with the increased severity of arthritic pathogenesis found in KLF2^−/−^ mice, we induced differentiation of bone marrow-derived osteoclast precursor cells to osteoclasts harvested from the femurs of KLF2^−/−^ and KLF2^+/+^ mice after the induction of arthritis. Osteoclast cells were stained for TRAP on days 3 and 6 during the course of differentiation. Osteoclastic differentiation was remarkably higher in precursor cells isolated from KLF2^−/−^ mice compared to KLF2^+/+^ mice at any given day of differentiation (Figure 5A,B). The number of nuclei was significantly higher in differentiated osteoclasts derived from KLF2^−/−^ mice compared to KLF2^+/+^ mice. To determine osteoclast functionality, osteoclasts were differentiated and cultured on thin ivory slices. Bone resorption assays were performed using osteoclasts isolated from both KLF2 (+/+) and KLF2 (−/−) groups of animals. Ivory slices were stained with hematoxylin and analyzed for the resorbed pits on day 10 of differentiation. A larger number of resorbed pits were formed by osteoclasts isolated from KLF2 (−/−) mice compared to KLF2 (+/+) mice (Figure 5C). Evaluated values of pit area/high power field (HPF) are shown graphically (Figure 5D). Therefore, these results indicate that there was an abundance of mature functional osteoclasts in KLF2 knockout mice. As it is known that MMPs such as MMP9 and MMP13 are highly associated with osteoclast differentiation and maturity [37,47], we sought to determine their level of expression in the bone marrow cells of KLF2^−/−^ arthritic mice. We found markedly increased levels of MMP9 and MMP13 in KLF2^−/−^ mice compared to KLF2^+/+^ mice after induction of arthritis (Figure 5E, and Supplementary Figure S2). Altogether, these data provide evidence for increased inflammation and severity of arthritic pathogenesis in KLF2^−/−^ mice.

### 3.6. Expression of KLF2 in Monocytes after Arthritis Development in Mice

**Figure 6:**
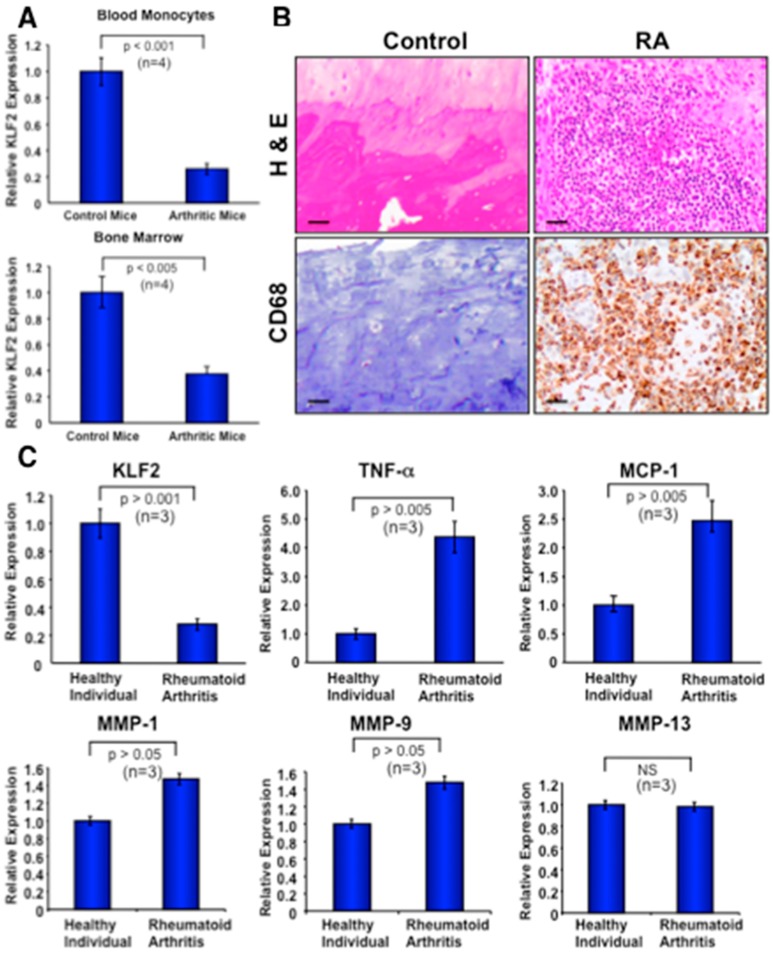
Expression of KLF2 is reduced in murine and human monocytes, and inflammatory molecules are elevated after arthritis development. (**A**) The expression level of KLF2 was measured in monocytes isolated from bone marrow and peripheral blood from mice using real-time PCR methods. GAPDH expression was used as an internal control for normalization of samples. (**B**) Human tissues affected by rheumatoid arthritis were stained to evaluate monocyte infiltration (right, lower panel) and H&E for morphology (right, upper panel); these were compared with the non-arthritic respective controls (left panels). (**C**) Quantitative real-time PCR was performed to determine relative expression of various arthritis-related factors in monocytes isolated from peripheral blood of rheumatoid arthritis patients as well as healthy controls.

We further investigated whether K/BxN serum-induced arthritis exerts any effect on the expression level of KLF2 in the monocytes of bone marrow or peripheral blood in C57/BL6 mice. To test this, we induced arthritis using K/BxN serum, and after 7 days isolated monocytes from the bone marrow and peripheral blood. RT-PCR analysis was performed on the extracted RNA of the harvested monocytes. Induced arthritic mice, which developed macroscopic symptoms of arthritis, were considered, and littermate controls without arthritis induction were considered as a control for comparison. Real-time PCR analysis revealed a significantly reduced level of KLF2 expression in monocytes either isolated from bone marrow (*p* < 0.005) or peripheral blood (*p* < 0.001) compared to the respective monocytes isolated from control mice without arthritis (Figure 6A). The expression of GAPDH was used for normalization of samples.

### 3.7. Recruitment of Activated Monocytes in Human Arthritic Joints and Expression of Molecules in Peripheral Blood Monocytes

Immunohistochemical analysis was performed to investigate the recruitment of activated monocytes (CD68^+^) to the inflammatory sites of RA tissues. A significant number of CD68^+^ cells were observed in all RA tissues examined (Figure 6B, lower right panel). Activated monocytes were present in both the lining and sublining layers of the tissues where inflammation occurred. No staining of macrophages appeared in the control non-arthritic tissues (Figure 6B, lower left panel). H&E staining in arthritic tissue sections demonstrated infiltration of numerous cells into the joint tissue, and these findings were consistent with every RA patient’s samples tested (Figure 6B, upper right panel). On the other hand, a negligible presence of cellular infiltration, or none at all, was observed in healthy control human tissues. These data confirm the infiltration of activated monocytes in the inflammatory site of arthritic joints.

To further determine the relative expression of various arthritis-related factors in monocytes isolated from the peripheral blood of active RA patients, as well as from healthy controls, RT-PCR was performed. Human blood and tissue samples were obtained from the OSUMC clinic after the institutional review board (IRB) approval from The Ohio State University Wexner Medical Center (IRB # 201280195). RT-PCR analysis (Figure 6C) revealed a significantly decreased level of KLF2 expression in monocytes isolated from arthritic patients (*n* = 3) compared to healthy (*n* = 3) control samples (*p* ˃ 0.001). These data further corroborate the increased expression of inflammatory factors such as TNFα (*p* ˃ 0.005), MCP-1 (*p* ˃ 0.005), MMP1 (*p* ˃ 0.05), and MMP9 (*p* ˃ 0.05). However, there was no significant change in the expression level of MMP13 between arthritic samples and control samples (Figure 6C). In summary, these data confirm that the decreased level of KLF2 in arthritic monocytes and concomitantly increased levels of various inflammatory and arthritis-related molecules are associated with disease progression.

### 3.8. Mechanistic Regulation of MMP9 by KLF2 in Monocytes

**Figure 7:**
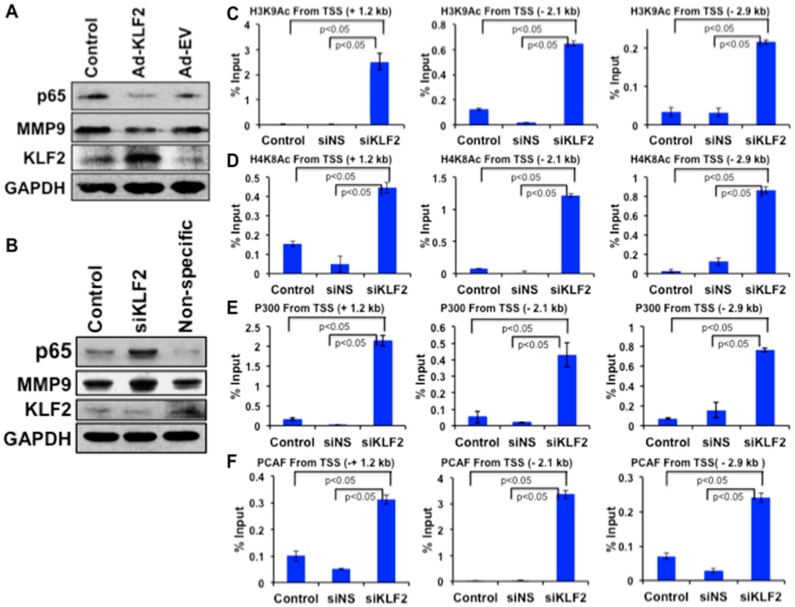
KLF2 inversely regulates MMP9 level, and KLF2 deficiency enriched active histone marks around transcriptional start site (TSS) of MMP9 in monocytes. (**A**) Western blot analysis of proteins after KLF2 overexpression. (**B**) Western blot analysis of proteins after KLF2 Knockdown. ChIP-RT-PCR analysis of histone acetylation marks and acetyltransferases on the promoter region of MMP9 using three different TSSs in monocytes after knockdown of KLF2 (siKLF2), non-specific knockdown (siNS) and control. (**C**) ChIP-RT-PCR analysis of H3K9Ac. (**D**) ChIP-RT-PCR analysis of H4K8Ac. (**E**) ChIP-RT-PCR analysis of p300. (**F**) ChIP-RT-PCR analysis of PCAF. Graphical results are means ± SEM from at least three independent experiments with triplicates.

KLF2 overexpression markedly reduced the levels of MMP9 and NF-κB (p65) proteins in monocytes (Figure 7A). Conversely, KLF2 knockdown significantly increased levels of MMP9 and p65 proteins in monocytes (Figure 7B). After establishing that MMP9 expression is inversely correlated with KLF2 expression, we further investigated the epigenetic mechanism of MMP9 regulation. From the University of California, Santa Cruz (UCSC) genome browser, we found that enrichment sites for epigenetic marks are located at +1.2 kb, −2.1 kb, and −2.9 kb upstream and downstream of the mouse MMP9 transcriptional start site (TSS). To understand the role of histone modifications in regulating MMP9 in the context of RA, the occupancy of histone acetylation marks (H3K9Ac, H4K8Ac) and histone acetyltransferase (HAT; P300, PCAF) were analyzed using the ChIP and RT-PCR methods. Analysis revealed that knockdown of KLF2 significantly increased the enrichment of active histone marks H3K9Ac and H4K8Ac (Figure 7C,D) and respective HAT enzymes P300 and PCAF (Figure 7E,F) at all three specific locations from the TSS of MMP9, compared to control and small interfering non-specific (siNS) transfected cells. These data indicate that absence of KLF2 promotes MMP9 expression via H3K9Ac and H4K8Ac occupancy through P300/PCAF enrichment.

## 4. Discussion

Krüppel-like factors (KLF) are a family of zinc-finger transcription factors that regulate the activation, differentiation, and function of various immune cells. KLF2, a member of this family, regulates quiescence, proliferation, differentiation, and survival of hematopoietic cells. KLF2 deficiency causes embryonic lethality due to leaky blood vessels and consequent hemorrhages [26]. KLF2 regulates T cells and also regulates myeloid cell activation and function [18], as well as myeloid cell polarization [22]. However, the regulatory role of KLF2 in the proinflammatory activation of monocytes and their differentiation in the context of K/BxN serum-induced RA pathogenesis has yet to be defined. To determine the critical importance of KLF2 in the pathological progression of RA, we used KLF2^+/−^ mice, and to confirm our findings we used monocyte-specific conditional KLF2 knockout mice in experimental RA induction studies.

The hallmarks of RA pathogenesis include pannus formation and synovial hyperplasia, mediated by infiltrating immune cells and proliferating fibroblasts. These events promote leukocyte recruitment, immune cell activation, and production of inflammatory mediators and proteinases, all of which eventually contribute to joint damage [48]. Our observations provide insights into the effects of the absence or presence of KLF2, including its significant influence on the manifestation of arthritic inflammatory swelling in mouse paws and ankle joints. The morphological appearance of mouse joint inflammation with progressively severe arthritis in KLF2^+/−^ mice revealed the inhibitory role of KLF2 in arthritic induction, which is further supported by the finding that inflammation was increased in KLF2 hemizygous mice (Figure 1). In RA, soft tissue swelling along with fluid exudation and cellular influx in the synovium is caused by inflammatory mediators mostly secreted by immune cells [49]. Our results showed that K/BxN serum-induced arthritis caused soft tissue swelling in arthritic mice, which was further assessed by analysis of histological evidence. Histological assessment revealed that a higher degree of inflammatory cell infiltration, along with damage to cartilage and bone tissue, were more prominent in the KLF2^+/−^ mice than in KLF2^+/+^ mice (Figure 1), which is consistent with our previous findings using an methylated bovine serum albumin (mBSA)- and IL-1β-mediated arthritis induction model [19].

Monocytes and macrophages are major sources of proinflammatory cytokines, including TNF-α, IL-6, IL-1, various chemokines, and MMPs, which play crucial roles in endothelial cell activation, additional recruitment of leukocytes to the inflamed joint site, acute phase reactions, osteoclastic differentiation, and cartilage and bone damage [48,50,51,52]. Inhibiting osteoclastogenesis, therefore, is an important goal for therapeutic intervention, since bone erosion causes irreversible structural damage associated with loss of joint function [48]. We found an increased level of osteoclastic differentiation of bone marrow osteoclast precursor cells from KLF2^+/−^ mice compared to KLF2^+/+^ mice (Figure 2), indicating that KLF2 has a crucial role in the prevention of osteoclastic differentiation. It is evident that proinflammatory factors promote osteoblast stimulation that facilitates osteoclast-mediated bone-resorbing activities [53]. In addition, it has been demonstrated that osteoclast formation is modulated by these inflammatory factors in bone marrow cells in vitro via up-regulation of RANKL and down-regulation of osteoprotegerin, the decoy receptor for RANKL [54]. RANKL could be substituted by TNF-α, which also mediates osteoclast formation from mouse bone marrow cells [55,56]. The cytokine IL-6 is also known to promote the development of osteoclast progenitor cells and stimulate osteoclast formation [57]. On the other hand, it has been shown that IL-l plays a crucial role in tissue damage by monocytes and macrophages [58]. This explains our finding of modest but significant elevated expression levels of IL-1, IL-6, CCL3, and MCP1 in the bone marrow cells of KLF2 heterozygous mice after induction of arthritis (Figure 3), which were associated with the formation of marginal erosions found in KLF2^+/−^ mice. These results indicate that KLF2 not only plays a critical role in inflammation but also plays a regulatory role in osteoclast differentiation. Conversely, the observation of reduced levels of IL-10 in bone marrow of KLF2^+/−^ mice also supports the regulatory role of KLF2 in inflammation and cytokine secretion from monocytes (Figure 3). Furthermore, we found elevated levels of MMPs in KLF2^+/−^ mice (Figure 3), which have a direct correlation with joint destruction in arthritic mice [16]. Our findings are in agreement with the previous observations, especially the reduction of KLF2 in mice resulted in enhanced production of MMPs in mice, particularly MMP9 (Figure 3).

The regulatory role played by KLF2 in the activation, differentiation, and function of monocytes was further confirmed using monocyte-specific conditional KLF2 knockout mice (in which ~90% reduction of KLF2 expression was observed) in a K/BxN serum-induced arthritis model. Severe inflammation, joint destruction, and increased expression of inflammatory genes in bone marrow cells were confirmed in KLF2 knockout mice, similar to the findings in KLF2^+/−^ mice (Figure 4). The occurrence of high osteoclast differentiation of osteoclastic precursor cells and increased levels of expression of MMPs, particularly MMP9, was correlated with the increased bone destruction found in KLF2 deficient mice (Figure 5). The role of KLF2 in monocytes was further confirmed in immunocompetent mice (C57BL/6 background) in which KLF2 level was significantly reduced in monocytes isolated from peripheral blood and bone marrow after induction of K/BxN serum-induced arthritis (Figure 6A). These findings provide evidence for the first time that KLF2 critically regulates K/BxN serum-induced arthritic severity by controlling monocyte activation and function and monocyte-mediated osteoclastic differentiation in K/BxN serum-induced arthritis.

Monocytes are known to be activated in RA patients [59], and CD68 is a useful marker for the various cells of the macrophage lineage, including monocytes and osteoclasts [60]. Activated monocytes infiltrate into the arthritic site. Therefore, infiltration of activated monocytes in human arthritic tissues are correlated with the severity of the disease. We also found that CD68^+^ monocytes were recruited to the arthritic site in the human tissue samples (Figure 6B). However, it was interesting to observe that the downregulation of KLF2 expression and concomitant upregulation of several inflammatory markers, including TNFα, MCP1, MMP1, MMP9, and MMP13, in primary monocytes isolated from the peripheral blood of patients with active RA (Figure 6C) established the inverse correlation between KLF2 levels and severe arthritic induction in humans. We have previously shown that KLF2 mechanistically regulates inflammatory mediators associated with monocyte activation and function through transcriptional factors, including NFκB and AP-1, via direct interaction with PCAF [18]. An imbalance in the interplay between pro- and anti-inflammatory genes can give rise to RA pathogenesis. Our systematic examination demonstrated that MMP9 and NF-κB are highly expressed during inflammatory responses (Figure 3 and Figure 7). We next demonstrated that overexpression of KLF2 decreased the levels of MMP9 and NF-κB; conversely, knockdown of KLF2 increased the levels of MMP9 and NF-κB in monocytes (Figure 7A,B). MMP9 transcription is sensitive to binding of epigenetic modulators like P300/CBP in the promoter region [61]. Studies suggest that most inflammatory genes in macrophages are enriched by the presence of H4 acetylation and H3K9/K14Ac marks that carry the signal of actively transcribed genes [29]. Our findings showed that KLF2 represents an intra-molecular mechanism of inflammatory gene regulation of MMP9. It could be possible that KLF2 regulates inflammatory genes through epigenetic mechanisms. Our ChIP analysis revealed that the decreased expression of KLF2 is responsible for the enrichment of H3K9Ac and H4K8Ac around the transcriptional start site (TSS) of the MMP9 promoter (Figure 7C,D) establishing the molecular basis of KLF2 and MMP9 crosstalk. To confirm that P300 and PCAF are required for H3K9Ac and H4K8Ac in monocyte cells, we performed further ChIP analyses for P300 and PCAF. We demonstrated that P300 and PCAF occupancy increased significantly, along with H3K9Ac and H4K8Ac marks (Figure 7E,F), confirming the regulatory role of KLF2 for the recruitment of P300/PCAF to the established H3K9Ac and H4K8Ac marks of MMP9. To the best of our knowledge, this is the first report documenting the role of KLF2 in epigenetic regulation of MMP9.

## 5. Conclusions

In summary, in this paper we have provided evidence that K/BxN serum-induced arthritic inflammation and pathogenesis, osteoclastic differentiation of monocytes, inflammatory cytokines and factors are significantly elevated both in KLF2 hemizygous mice and in monocyte-specific conditional KLF2 knockout mice, indicating the importance of myeloid KLF2 in RA pathogenesis. Induction of RA in mice resulted in a decreased level of KLF2 in monocytes. Human RA patients also showed a decreased level of KLF2 and concomitant increased levels of inflammatory factors and MMPs in peripheral blood monocytes, as well as increased migration of activated monocytes to the RA sites. Mechanistically, overexpression of KLF2 decreased the level of MMP9; conversely, knockdown of KLF2 increased MMP9 in monocytes along with enrichment of active histone marks, as well as histone acetyltransferases on the MMP9 promoter region. In conclusion, our data confirm the critical regulatory role of myeloid KLF2 in K/BxN serum-induced RA pathogenesis. These findings provide an opportunity for future development of RA therapeutics by targeting the KLF2 molecule.

## Supplementary Materials

The following are available online at https://www.mdpi.com/2073-4409/8/8/908/s1; Figure S1. Mouse UCSC genome browser analysis, Figure S2. Density measurements of the western blot data shown in Figure 5E. Table S1. Primer sequences used for chromatin immunoprecipitation.
