# Supplementary material for: Myeloid Krüppel-Like Factor 2 Critically Regulates K/BxN Serum-Induced Arthritis
Source: Cells. 2019 Aug 16;8(8):908. doi: 10.3390/cells8080908 (PMC6721677; doi:10.3390/cells8080908)
Supplement: Supplementary file 1 [file cells-08-00908-s001.pdf]

Supplemental Figure 1. Mouse UCSC genome browser analysis.
UCSC genome browser track of mouse genome analyzed and three genomic sites were chosen to analyze by ChIP-PCR. The red bar indicates the locations that analyzed by ChIP-RT-PCR.

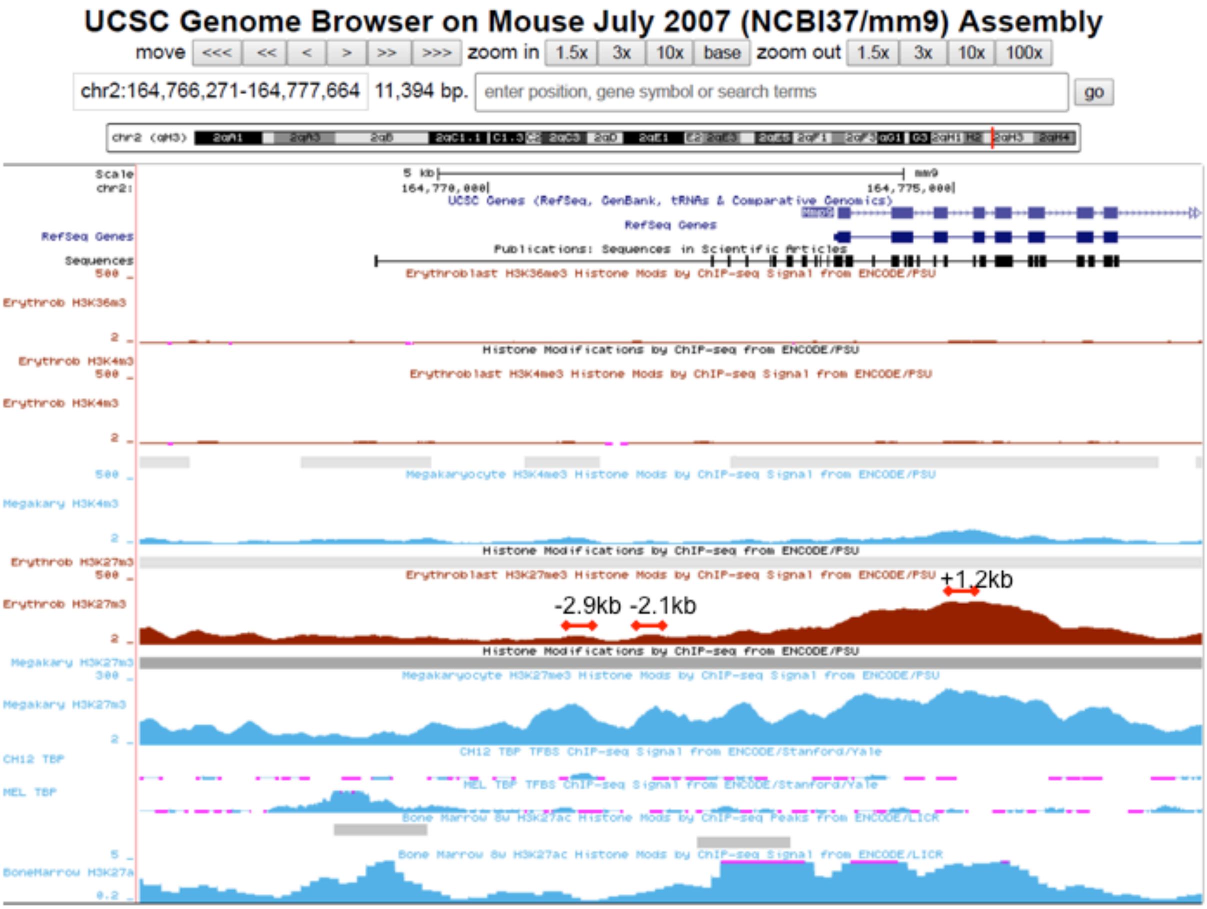

Supplemental Figure 2. Density measurement of western blot data shown in Figure 5E.

Density measurements of the western blot data shown in Fig. 5E

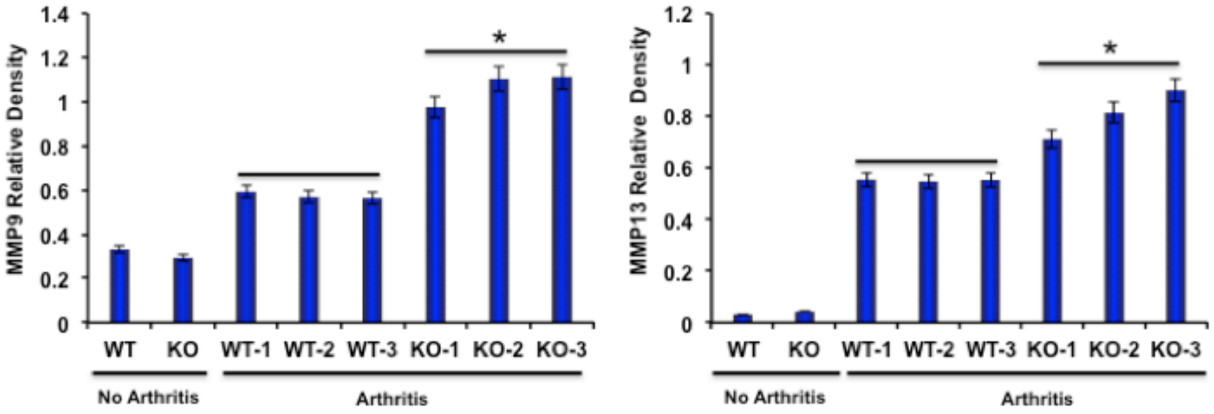

Supplemental Table I. Primer sequences used for chromatin immunoprecipitation assays.

Table I. Primer sequences used for chromatin immunoprecipitation assays

Name	Forward primer	Reverse primer	Product Length (bp)	Genome Region From TSS
chr2:164775019-164775122	5'-ATCTGTCCGCTCTTATGCCTG-3'	5'-TTGAGAAGAGGTGACTGCGAC-3'	103	+1.2 kb
chr2:164771647-164771863	5'-TCACGGGGACCTGAGATCAT-3'	5'-TGACCCACTTGGCCATTTCC-3'	238	-2.1 kb
chr2:164770742-164770948	5'-ATAGTGGGACCCTGCCTCAA -3'	5'-ACGTGGTGTGTCCAGTCT-3'	205	-2.9 kb
